# Ocurrence dataset of birds in university campuses of Nanjing, Juangsu Province China

**DOI:** 10.3897/BDJ.12.e126064

**Published:** 2024-05-27

**Authors:** Lijun Xiao, Ding Chen, Zheping Xu, Yong Zhang

**Affiliations:** 1 Co-Innovation Center for Sustainable Forestry in Southern China, Nanjing, China Co-Innovation Center for Sustainable Forestry in Southern China Nanjing China; 2 College of Life Sciences, Nanjing Forestry University, Nanjing, China College of Life Sciences, Nanjing Forestry University Nanjing China; 3 Chinese Academy of Sciences, Beijing, China Chinese Academy of Sciences Beijing China

**Keywords:** University area, avian community composition, urbanisation

## Abstract

**Background:**

The protection of urban biodiversity plays a critical role in maintaining urban ecological security. As a unique taxon of urban wildlife, birds have been intensively used as an indicator of our surrounding environment. Although the diversity of urban bird species has received increasing attention, present studies are mainly focused on urban parks. As one of the important components of the urban ecosystem, the university campus also plays a critical role in maintaining bird diversity. Due to the lack of observation data, it is a challenge to evaluate the impact of the environmental conditions on campus bird diversity. Given the most concentrated higher education resources in China, Nanjing has a large number of universities, covering a wide area of the urban landscape. The landscape of these universities usually has a high percentage of green space as well as waterbodies, which is conducive to attracting various bird species to inhabit. Here, we conducted a one-year bird survey from January 2019 to December 2019 in 12 universities in Nanjing and provided an occurrence dataset containing detailed species and geographical information, providing a good source to study the ecological and anthropogenic factors on urban bird diversity on a local and larger scale.

**New information:**

This dataset represents the first annual record of birds publicly released by 12 universities in Nanjing, Jiangsu Province. It includes classification information, population, distribution and survey details. All data have been published on GBIF.

## Introduction

The impact of human activities on biodiversity is profound and complex (Xiang et al. 2023). Since humans migrated out of Africa and dispersed to other continents, successive waves of species extinctions have been triggered (Cooke et al. 2023). There is a substantial body of research indicating that, since the Late Pleistocene, approximately 1300–1500 bird species have become extinct globally, with human activities (e.g. habitat destruction, climate change and illegal hunting) generally considered as one of the primary driving factors (Cooke et al. 2023). This anthropogenic extinction is not random; rather, it leads to the loss of species with unique traits or evolutionary histories. Such extinction may result in the convergence of functional traits amongst species in the community and a closer genetic relationship, thereby reducing the phylogenetic and functional diversity in the community and leading to homogenisation. (Purvis et al. 2000).

Rapid urbanisation has provided great needs for humans, while also posing new crises for other living organisms (Storch et al. 2022). Urbanisation processes that convert the original natural landscape into a human-dominated scene generally have a negative impact on biodiversity (Ibáñez-Álamo et al. 2020). In urban landscapes, the factors affecting urban bird diversity and community structure are tangled, involving both natural and anthropogenic elements. Amongst them, factors caused by human activities, such as land cover, habitat fragmentation and population density, greatly affect bird distribution, abundance and diversity (Zhang et al. 2018, Prakash and Verma 2022). Different bird species have significant preferences for different types of land cover and with the acceleration of the urbanisation process, the land cover changes, resulting in a filtering effect on the distribution of birds (Sun et al. 2022). Another study showed that grasslands, buildings and water areas are the primary land-cover types affecting bird diversity (Zhao et al. 2023). In addition, urban population size also had a negative correlation with bird richness (Gagné et al. 2016).

University campuses are vital components of urban ecosystems, which contain a large proportion of green spaces (Liu et al. 2021, Sanllorente et al. 2023). The development of higher education in China has spurred a wave of new campus construction, with university campuses now covering more than 620 km^2^ of urban area and many campuses exceeding 100 hectares (Liu et al. 2021). Nanjing City, a national centre of scientific research and education in China, has established multiple university towns like Xianlin, Jiangning and Pukou, which provide a great opportunity for urban bird studies.

A study comparing bird diversity between university campuses and other urban areas found the number of bird species was significantly higher on the campus (almost 50% more) in Spain. Additionally, the study also pointed out that the number of protected bird species on university campuses is almost twice that of other urban areas (Sanllorente et al. 2023). Another study also found campuses are crucial refuges for urban birds, with an average of 66 species per campus, including some endangered species (Liu et al. 2021). These findings align with the common prediction that university campuses act as special areas with high bird richness in the urban environment.

As a vital indicator of urban environments, the diversity of birds can reflect the health of the ecological environment directly or indirectly. Long-term monitoring of bird diversity in specific areas is, therefore, needed to understand ecosystem trends (Tong et al. 2023). So far, there is still a lack of campus bird survey data in Nanjing, impeding the related studies both locally and on a large scale. Here, we conducted a one-year bird survey across 12 universities in Nanjing, Jiangsu Province and reported an occurrence dataset containing detailed species and geographical information, providing comprehensive data for further research on campus and urban bird studies.

## Sampling methods

### Sampling description

We selected 12 university campuses located in both the central old city area of Nanjing and the sub-urban Xianlin University Town. From January 2019 to December 2019, monthly bird surveys were conducted on each campus using the transect method. Within each campus, two fixed transects were established to cover various habitat types as much as possible, including waterbodies, grasslands, woodlands, teaching areas and sports fields. The length of the transects varied from 0.5 to 2 km, depending on the size of the campus and they were designed to traverse the entire campus. Surveys were conducted on clear days without rain, snow or strong winds, between 07:00 h and 10:00 h in the morning and 16:00 h and 19:00 h in the afternoon. Surveyors walked along the transects at a speed of about 3 km per hour, observing and recording birds using 10 × 40 binoculars (Zhang et al. 2021). For flying birds, only those flying in the opposite direction to the movement of the surveyors were recorded, while those flying from back to front were not included. The species and numbers of birds along each transect were recorded during the surveys. For large flocks, a group counting method was used. Species identification was primarily based on "A Checklist on the Classification and Distribution of the Birds of China (Fourth Edition)" (Zheng 2023).

## Geographic coverage

### Description

**Figure 1.**
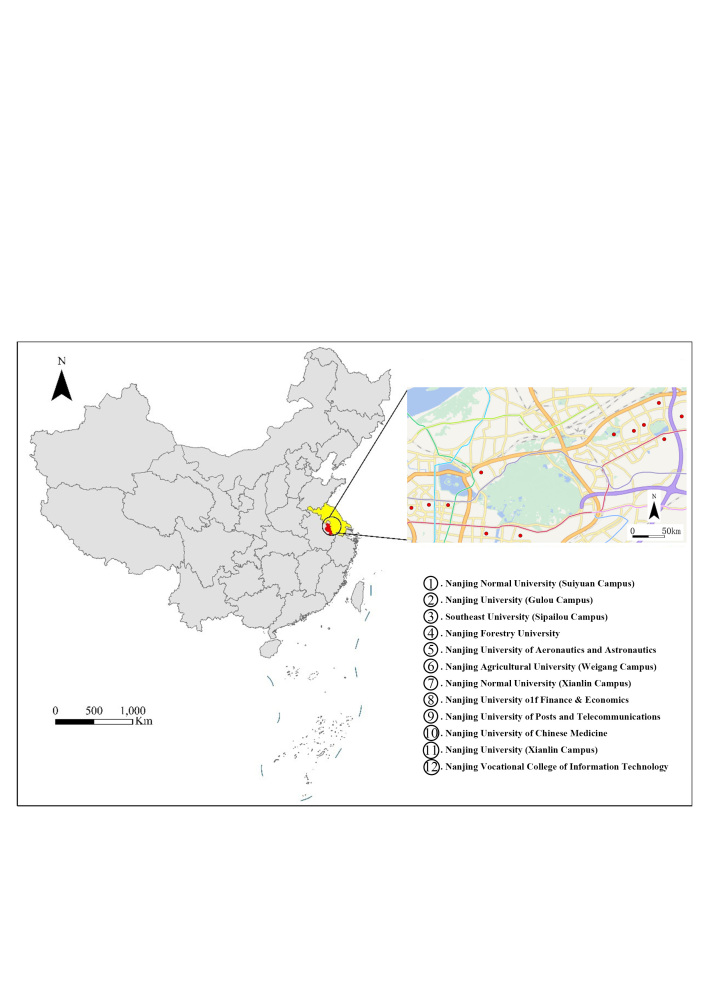
The geographical location of 12 university campuses in Nanjing, China.

Nanjing is located in the eastern part of China, in the middle reaches of the lower Yangtze River and features a subtropical monsoon climate with hot and rainy summers and cold, less rainy winters. It is influenced by the East Asian summer monsoon and the Indian summer monsoon, with most precipitation concentrated in the summer. The survey area encompasses 12 universities, all located in Nanjing, Jiangsu Province (Fig. 1). Specifically, Southeast University (Sipailou Campus), Nanjing Forestry University and Nanjing Agricultural University (Weigang Campus) are in Xuanwu District. Nanjing University of Aeronautics and Astronautics is in Qinhuai District. Nanjing University (Gulou Campus) and Nanjing Normal University (Suiyuan Campus) are in Gulou District. Nanjing University of Finance & Economics, Nanjing University (Xianlin Campus), Nanjing Normal University (Xianlin Campus), Nanjing Vocational College of Information Technology, Nanjing University of Posts and Telecommunications and Nanjing University of Chinese Medicine are in Qixia District.

### Coordinates

32.03°N and 32.13°N Latitude; 118.76°E and 118.96°E Longitude.

## Taxonomic coverage

### Description

The occurrence dataset recorded a total of 11,518 individual birds, belonging to 12 orders, 32 families and 66 species. Amongst these, the Passeriformes had the highest number of species, with 40 species accounting for 60.61% of the total recorded bird species, followed by the Ciconiiformes with five species, making up 7.58%. In terms of individual bird counts, the Passeriformes also had the highest number, totalling 9,933 individuals, which represents 86.24% of the total bird count, with Eurasian Tree Sparrow (*Passermontanus*) being the most numerous at 1,577 individuals. The Columbiformes ranked second in individual counts with 1,046 individuals, accounting for 9.08% of the total. From the perspective of bird ecological habits, resident species were the most numerous, including *P.montanus*, *Gallinulachloropus*, *Streptopeliaorientalis*, *Pycnonotussinensis*, *Spilopeliachinensis* etc. There were 26 migrant species, including *Ardeolabacchus*, *Bubulcusibis*, *Cecropisdaurica*, *Anaszonorhyncha*, *Tringaochropus* etc., indicating that the bird population on Nanjing's university campuses was primarily composed of resident birds, with both species counts and individual numbers significantly higher than those of migratory birds (judging resident and migratory birds was based on year-round surveys). For different campuses, Nanjing Agricultural University (Weigang Campus) recorded 21 species with 647 individuals, Nanjing Forestry University had 28 species with 1,369 individuals, Nanjing Normal University (Suiyuan Campus) found 30 species with 463 individuals, Nanjing Normal University (Xianlin Campus) recorded 29 species with 730 individuals, Nanjing University (Gulou Campus) had 20 species with 580 individuals, Nanjing University (Xianlin Campus) recorded 34 species with 1,583 individuals, Nanjing University of Aeronautics and Astronautics found 33 species with 466 individuals, Nanjing University of Chinese Medicine recorded 36 species with 1,850 individuals, Nanjing University of Finance & Economics had 35 species with 907 individuals, Nanjing University of Posts and Telecommunications recorded 41 species with 1,601 individuals, Nanjing Vocational College of Information Technology found 32 species with 998 individuals and Southeast University (Sipailou Campus) recorded 20 species with 324 individuals. Additionally, amongst the recorded birds, *Milvusmigrans* is a nationally protected wildlife species in China and, according to the IUCN Red List of Threatened Species, all 66 recorded bird species are classified as Least Concern (LC) (Table 1).

## Temporal coverage

### Notes

The specific dates of this period were: 2019-01-22~2019-01-24; 2019-02-27~2019-02-28; 2019-03-29~2019-03-30; 2019-04-27~2019-04-29; 2019-05-22~2019-05-23; 2019-06-19~2019-06-23; 2019-07-13~2019-07-23; 2019-08-27~2019-08-31; 2019-09-25~2019-09-29; 2019-10-8~2019-10-30; 2019-11-18~2019-11-29; 2019-12-2~2019-12-29.

## Usage licence

### Usage licence

Other

### IP rights notes

Creative Commons Attribution Non-Commercial (CC-BY-NC) 4.0 License

## Data resources

### Data package title

Occurrence dataset of birds in 12 Universities in Nanjing, China

### Resource link

https://doi.org/10.15468/xk82yf

### Alternative identifiers

http://www.gbifchina.org.cn/resource?r=nanjingcampusbird

### Number of data sets

1

### Data set 1.

#### Data set name

Occurrence dataset of birds in 12 Universities in Nanjing, China

#### Data format

Darmin Core Archive format

#### Download URL

http://www.gbifchina.org.cn/archive.do?r=nanjingcampusbird

#### Description

This dataset originates from a bird survey conducted at 12 universities in Nanjing, Jiangsu Province, China, during the period from 2019 to 2020 (Chen et al. 2024). It contains detailed species and geographic information.

**Data set 1.** 

Column label	Column description
eventID (Event core, Occurrence extention)	An identifier for the set of information associated with an Event (something that occurs at a place and time).
eventDate (Event core)	The date-time or interval during which an Event occurred. For occurrences, this is the date-time when the event was recorded. Not suitable for a time in a geological context.
samplingProtocol (Event core)	The names of, references to, or descriptions of the methods or protocols used during an Event.
samplingEffort (Event core)	The amount of effort expended during an Event.
sampleSiveValue (Event core)	A numeric value for a measurement of the size (time duration, length, area or volume) of a sample in a sampling event.
sampleSizeUnit (Event core)	The unit of measurement of the size (time duration, length, area, or volume) of a sample in a sampling event.
decimalLongitude (Event core, Ocurrence Extension)	The geographic longitude (in decimal degrees, using the spatial reference system given in geodeticDatum) of the geographic centre of a Location.
decimalLatitude (Event core, Ocurrence Extension)	The geographic latitude (in decimal degrees, using the spatial reference system given in geodeticDatum) of the geographic centre of a Location.
geodeticDatum (Event core)	The ellipsoid, geodetic datum or spatial reference system (SRS), upon which the geographic coordinates given in decimalLatitude and decimalLongitude are based.
countryCode (Event core)	The standard code for the country in which the Location occurs.
country (Event core)	The name of the country or major administrative unit in which the Location occurs.
stateProvince (Event core)	The name of the next smaller administrative region than country (state, province, canton, department, region etc.) in which the Location occurs.
county (Event core)	The full, unabbreviated name of the next smaller administrative region than stateProvince (county, shire, department etc.) in which the Location occurs.
locality (Event core, Occurrence Extention)	The specific description of the place.
locationID (Event core)	An identifier for the set of location information (data associated with dcterms:Location). May be a global unique identifier or an identifier specific to the dataset.
parentEventID (Event core)	An identifier for the broader dwc:Event that groups this and potentially other dwc:Events.
coordinateUncertaintyInMetres (Event core)	The horizontal distance (in metres) from the given decimalLatitude and decimalLongitude describing the smallest circle containing the whole of the Location. Leave the value empty if the uncertainty is unknown, cannot be estimated or is not applicable (because there are no coordinates). Zero is not a valid value for this term.
occurrenceID (Occurrence Extension)	An identifier for the bird occurrence.
basisOfRecord (Occurrence Extension)	The specific nature of the data record.
recordedBy (Occurrence Extension)	A list (concatenated and separated) of names of people, groups or organisations responsible for recording the original Occurrence. The primary collector or observer, especially one who applies a personal identifier (recordNumber), should be listed first.
individualCount (Occurrence Extension)	The number of individuals present at the time of the Occurrence.
organismQuantity (Occurrence Extension)	A number or enumeration value for the quantity of organisms.
organismQuantityType (Occurrence Extension)	The type of quantification system used for the quantity of organisms.
occurrenceStatus (Occurrence Extension)	A statement about the presence or absence of a Taxon at a Location.
scientificName (Occurrence Extension)	The full scientific name.
scientificNameAuthorship (Occurrence Extension)	The authorship information for the dwc:scientificName formatted according to the conventions of the applicable dwc:nomenclaturalCode.
kingdom (Occurrence Extension)	The full scientific name of the kingdom in which the taxon is classified.
phylum (Occurrence Extension)	The full scientific name of the phylum in which the taxon is classified.
class (Occurrence Extension)	The full scientific name of the class in which the taxon is classified.
order (Occurrence Extension)	The full scientific name of the order in which the taxon is classified.
family (Occurrence Extension)	The full scientific name of the family in which the taxon is classified.
genus (Occurrence Extension)	The full scientific name of the genus in which the dwc:Taxon is classified.
taxonRank (Occurrence Extension)	The taxonomic rank of the most specific name in the scientificName as it appears in the original record.
ownerInstitutionCode (Occurrence Extension)	The name (or acronym) in use by the institution having ownership of the object(s) or information referred to in the record.
dynamicProperties (Occurrence Extension)	A list of threatened level of about the record according to The IUCN Red List of Threatened Species (Version 2023-1). Meant to provide a mechanism for structured content.

## Figures and Tables

**Table 1.** The list of bird species found in studied campuses in Nanjing.

Rank	Order	Family	Scientific Name	The number of individual birds observed
1	Galliformes	Phasianidae	*Bambusicolathoracicus* (Temminck, 1815)	9
2	Galliformes	Phasianidae	*Phasianuscolchicus* Linnaeus, 1758	1
3	Anseriformes	Anatidae	*Anaszonorhyncha* Swinhoe, 1866	41
4	Podicipediformes	Podicipedidae	*Tachybaptusruficollis* (Pallas, 1764)	74
5	Columbiformes	Columbidae	*Columbalivia* J.F.Gmelin, 1789	38
6	Columbiformes	Columbidae	*Streptopeliaorientalis* (Latham, 1790)	422
7	Columbiformes	Columbidae	*Streptopeliachinensis* (Scopoli, 1786)	586
8	Cuculiformes	Cuculidae	*Eudynamysscolopaceus* (Linnaeus, 1758)	8
9	Cuculiformes	Cuculidae	*Hierococcyxsparverioides* (Vigors, 1832)	11
10	Cuculiformes	Cuculidae	*Cuculusmicropterus* Gould, 1838	3
11	Gruiformes	Rallidae	*Gallinulachloropus* (Linnaeus, 1758)	274
12	Charadriformes	Charadriidae	*Vanelluscinereus* (Blyth, 1842)	1
13	Charadriformes	Scolopacidae	*Tringaochropus* Linnaeus, 1758	6
14	Pelecaniformes	Ardeidae	*Nycticoraxnycticorax* (Linnaeus, 1758)	15
15	Pelecaniformes	Ardeidae	*Butoridesstriata* (Linnaeus, 1758)	2
16	Pelecaniformes	Ardeidae	*Ardeolabacchus* (Bonaparte, 1855)	10
17	Pelecaniformes	Ardeidae	*Bubulcusibis* (Linnaeus, 1758)	4
18	Pelecaniformes	Ardeidae	*Egrettagarzetta* (Linnaeus, 1766)	34
19	Accipitriformes	Accipitridae	*Milvusmigrans* (Boddaert, 1783)	5
20	Coraciiformes	Coraciidae	*Eurystomusorientalis* (Linnaeus, 1766)	2
21	Coraciiformes	Alcedinidae	*Alcedoatthis* (Linnaeus, 1758)	3
22	Coraciiformes	Alcedinidae	*Cerylerudis* (Linnaeus, 1758)	8
23	Piciformes	Picidae	*Picumnusinnominatus* Burton, 1836	1
24	Piciformes	Picidae	*Dendrocoposcanicapillus* (Blyth, 1845)	14
25	Piciformes	Picidae	*Dendrocoposmajor* (Linnaeus, 1758)	8
26	Piciformes	Picidae	*Picuscanus* J.F.Gmelin, 1788	5
27	Passeriformes	Dicruridae	*Dicrurusmacrocercus* Vieillot, 1817	4
28	Passeriformes	Laniidae	*Laniuscristatus* Linnaeus, 1758	41
29	Passeriformes	Laniidae	*Laniusschach* Linnaeus, 1758	67
30	Passeriformes	Corvidae	*Cyanopicacyanus* (Pallas, 1776)	1,571
31	Passeriformes	Corvidae	*Urocissaerythroryncha* (Boddaert, 1783)	17
32	Passeriformes	Corvidae	*Dendrocittaformosae* Swinhoe, 1863	2
33	Passeriformes	Corvidae	*Picapica* (Linnaeus, 1758)	432
34	Passeriformes	Paridae	*Pardaliparusvenustulus* (Swinhoe, 1870)	1
35	Passeriformes	Paridae	*Paruscinereus* Vieillot, 1818	156
36	Passeriformes	Hirundinidae	*Hirundorustica* Linnaeus, 1758	78
37	Passeriformes	Hirundinidae	*Cecropisdaurica* (Laxmann, 1769)	68
38	Passeriformes	Pycnonotidae	*Spizixossemitorques* Swinhoe, 1861	20
39	Passeriformes	Pycnonotidae	*Pycnonotusjocosus* (Linnaeus, 1758)	2
40	Passeriformes	Pycnonotidae	*Pycnonotussinensis* (Gmelin, 1789)	1,365
41	Passeriformes	Phylloscopidae	*Phylloscopusproregulus* (Pallas, 1811)	20
42	Passeriformes	Phylloscopidae	*Phylloscopusinornatus* (Blyth, 1842)	26
43	Passeriformes	Cettiidae	*Horornisfortipes* Hodgson, 1845	4
44	Passeriformes	Aegithalidae	*Aegithalosglaucogularis* (Moore, 1855)	164
45	Passeriformes	Aegithalidae	*Aegithalosconcinnus* (Gould, 1855)	202
46	Passeriformes	Sylviidae	*Sinosuthorawebbiana* (Gould, 1852)	111
47	Passeriformes	Zosteropidae	*Zosteropssimplex* Swinhoe, 1861	6
48	Passeriformes	Leiothrichidae	*Garrulaxperspicillatus* (Gmelin, 1789)	217
49	Passeriformes	Sturnidae	*Acridotherescristatellus* (Linnaeus, 1758)	526
50	Passeriformes	Sturnidae	*Spodiopsarsericeus* (Gmelin, 1789)	130
51	Passeriformes	Sturnidae	*Spodiopsarcineraceus* (Temminck, 1835)	1,392
52	Passeriformes	Sturnidae	*Gracupicanigricollis* (Paykull, 1807)	28
53	Passeriformes	Turdidae	*Turdushortulorum* P.L.Sclater, 1863	15
54	Passeriformes	Turdidae	*Turdusmandarinus* Bonaparte, 1850	1,189
55	Passeriformes	Turdidae	*Turduseunomus* Temminck, 1831	5
56	Passeriformes	Muscicapidae	*Tarsigercyanurus* (Pallas, 1773)	4
57	Passeriformes	Muscicapidae	*Copsychussaularis* (Linnaeus, 1758)	91
58	Passeriformes	Muscicapidae	*Phoenicurusauroreus* (Pallas, 1776)	15
59	Passeriformes	Estrildidae	*Lonchurastriata* (Linnaeus, 1766)	6
60	Passeriformes	Passeridae	*Passermontanus* (Linnaeus, 1758)	1,577
61	Passeriformes	Motacillidae	*Motacillacinerea* Tunstall, 1771	1
62	Passeriformes	Motacillidae	*Motacillaalba* Linnaeus, 1758	186
63	Passeriformes	Motacillidae	*Anthushodgsoni* Richmond, 1907	14
64	Passeriformes	Fringillidae	*Fringillamontifringilla* Linnaeus, 1758	21
65	Passeriformes	Fringillidae	*Eophonamigratoria* Hartert, 1903	131
66	Passeriformes	Fringillidae	*Chlorissinica* (Linnaeus, 1766)	28
